# Odontoid process fractures: the role of the ligaments in maintaining stability. A biomechanical, cadaveric study

**DOI:** 10.1051/sicotj/2015011

**Published:** 2015-06-15

**Authors:** Oliver Richard Boughton, Jason Bernard, Matthew Szarko

**Affiliations:** 1 St George’s, University of London Tooting, London SW17 0RE UK; 2 St George’s, Healthcare NHS Trust Tooting, London SW17 0RE UK

**Keywords:** Odontoid process, Odontoid process fracture, Ligament, Biomechanical, Cadaveric

## Abstract

*Aims*: We wished to investigate the role of the cervical ligaments in maintaining atlantoaxial stability after fracture of the odontoid process.

*Methods*: We dissected eight fresh-frozen cadaveric cervical spines to prepare the C1 and C2 vertebrae for biomechanical analysis. The C1 and C2 blocks were mounted and biomechanical analysis was performed to test the stability of the C1-C2 complex after cutting the odontoid process to create an Anderson and D’Alonzo type II fracture then successive division of the atlantoaxial ligaments. Biomechanical analysis of stiffness, expressed as Young’s modulus, was performed under right rotation, left rotation and anterior displacement.

*Results*: The mean Young’s modulus in anterior displacement decreased by 37% when the odontoid process was fractured (*p* = 0.038, 95% confidence interval 0.04–1.07). The mean Young’s modulus in anterior displacement decreased proportionally (compared to the previous dissection) by the following percentages when the structures were divided: facet joint capsules (bilateral) 16%, ligamentum flavum 27%, anterior longitudinal ligament 10%. These differences did not reach statistical significance (*p* > 0.05).

*Discussion*: We have found that the odontoid process itself may account for up to 37% of the stiffness of the C1-C2 complex and that soft tissue structures account for further resistance to movement. We suggest magnetic resonance imaging (MRI) of the soft tissues in the acute setting of a minimally displaced odontoid process fracture to plan management of the injury. If the MRI determines that there is associated ligament injury it is likely that the fracture is unstable and we would suggest operative management.

## Introduction

Sixty percent of spinal injuries affect the cervical spine [1] and 9–20% of cervical spine injuries involve the axis (C2) [1–3]. There is a bimodal distribution with low-energy fractures in the elderly and high-energy fractures in young patients [2]. They can be devastating injuries with associated neurological injury in 2–27% of patients [2, 3] and the acute mortality rate is 2.4% [3]. Odontoid process fractures are the most common fractures of the axis [3]. Anderson and D’Alonzo [4] classified odontoid process fractures into three types in 1974. Type II odontoid process fractures are fractures through the waist of the odontoid process, between the level of the transverse ligament and C2 vertebral body [2, 4]. Type II odontoid process fractures have a one-year mortality rate of 18% in patients over 65 years of age [5].

The current management of type II odontoid process fractures is controversial [1]. Opinion is divided as to whether these fractures should be treated non-operatively (halo device or cervical collar) or operatively (anterior screw fixation or posterior C1-C2 fusion) [1]. A meta-analysis was performed of operative versus non-operative management of Anderson and D’Alonzo type II odontoid process fractures by Nourbakhsh et al. in 2009 [6]. They looked at the primary outcome measure of bone fusion after operative (either C1-C2 fusion or anterior screw fixation) versus non-operative management (halo vest immobilisation or cervical collar). They recommended operative treatment for older patients, in fractures with posterior displacement and when the displacement of the fracture is greater than 4–6 mm [6].

There is little known about the association of ligament injuries with odontoid process fractures. Greene et al. in 1994, in a study using a combination of computed tomography (CT) and magnetic resonance imaging (MRI), observed that ligament injuries could be detected using MRI and identified potential ligament injuries associated with odontoid process fractures that would alter the management of the condition [7]. Sasso in 2001 commented that C2 fractures “should not be considered isolated bony injuries” and that ligament injuries should be considered [8].

The aim of our study was to investigate the role of the atlantoaxial ligaments in maintaining atlantoaxial stability after Anderson and D’Alonzo type II odontoid process fractures. We investigated the biomechanics of the ligaments after type II fractures of the odontoid process. We used a biomechanical cadaveric study to investigate the ligaments. We measured stiffness of the C1-C2 complex during both anterior-posterior (AP) displacement and rotation, before and after odontoid fracture.

## Methods

We dissected eight fresh-frozen cadaveric spines, dissecting out the C1 and C2 vertebrae with all the ligaments between C1 and C2 left intact. The specimens were thawed before mechanical testing. Our aim was to investigate the contribution of each individual ligament and soft tissue structures within the C1-C2 complex to stability of the complex after the odontoid process was fractured.

**Figure 1.**
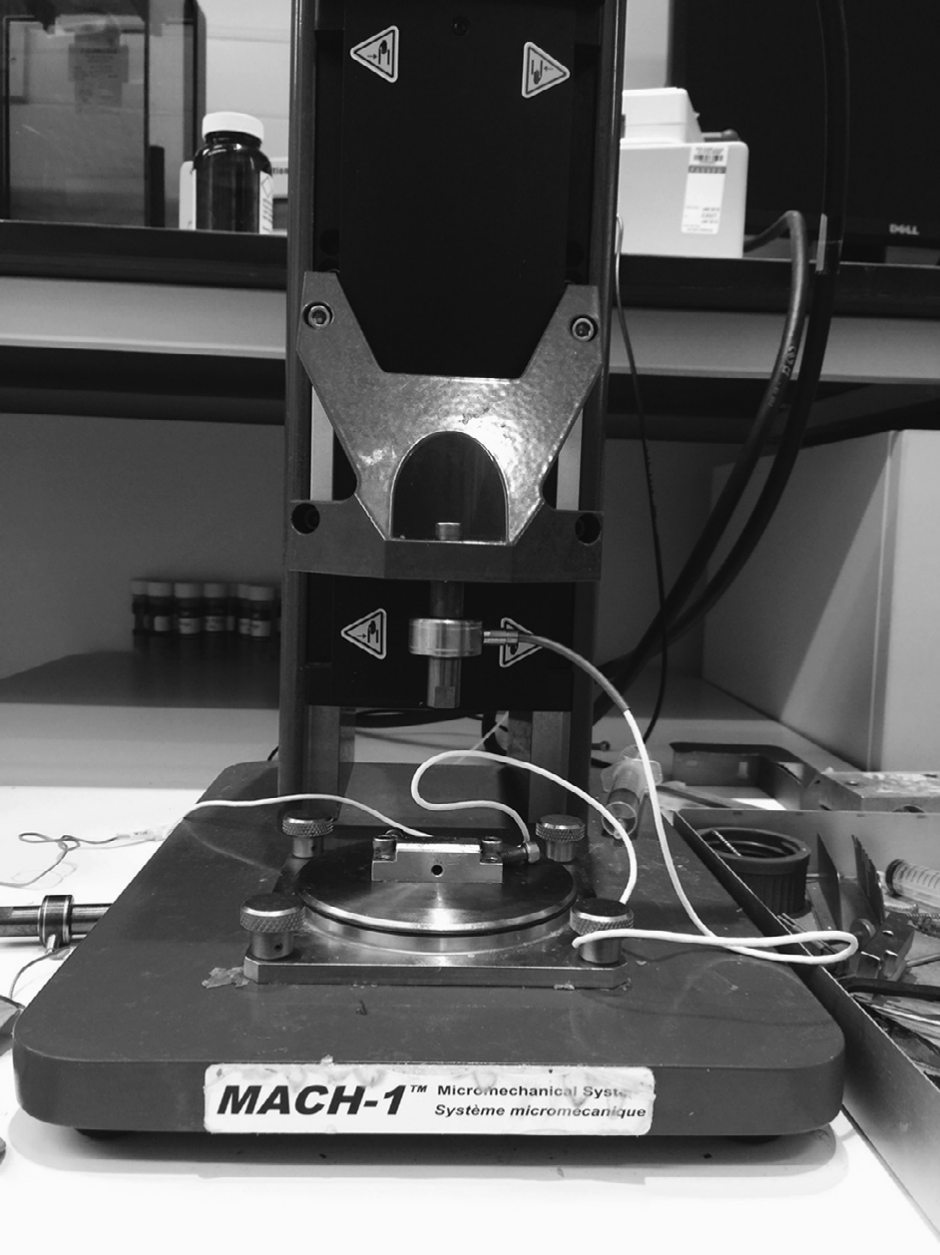
The MACH-1 Materials Testing Device, Biomomentum, Canada.

**Figure 2.**
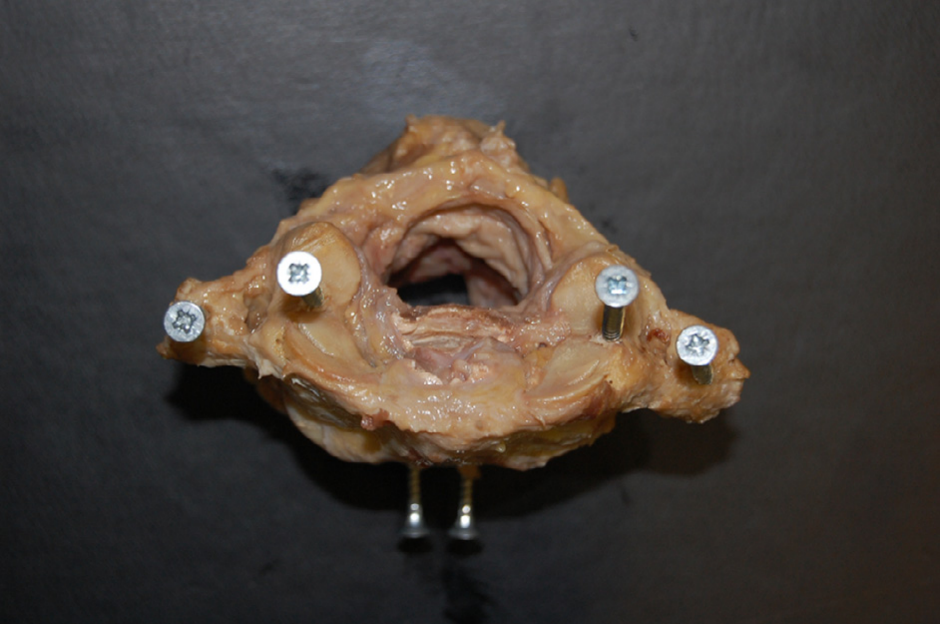
C1-C2 fresh-frozen cadaveric spine C1-C2 complex viewed from superior aspect. Two screws were inserted into the lateral masses, two screws were inserted into the transverse processes and two screws were inserted from anterior to posterior direction into the C2 vertebral body.

**Figure 3.**
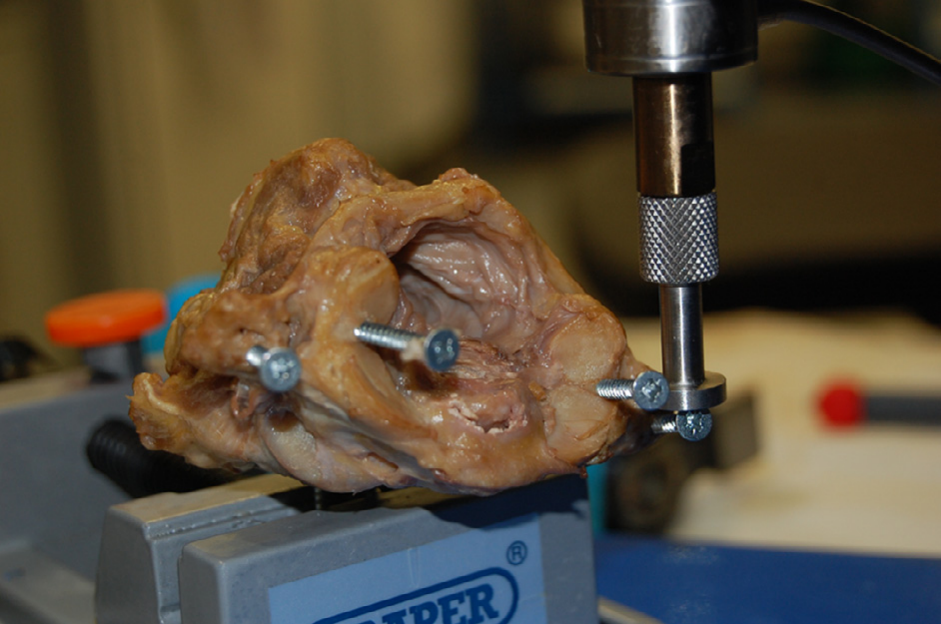
C1-C2 complex with lateral rotation testing being demonstrated.

Mechanical stability of the complex was assessed using a MACH-1 materials testing device, Biomomentum, Canada (Figure 1). To enable testing, six screws were inserted into each C1-C2 complex. Two screws were inserted from an anterior to posterior direction into the vertebral body of C2 (Figure 2). This enabled the C1-C2 complex to be held in a clamp. A screw was inserted vertically into the lateral mass of the atlas on either side. These lateral mass screws were used to support an aluminium bar that enabled the testing machine to test anterior-posterior (AP) displacement stability. A screw was then inserted vertically into the transverse process of the atlas on either side. These screws enabled the machine to test left and right rotational stability (Figure 3).

In determining the stability of the C1-C2 complex, it was determined that a 3 mm anterior displacement and a 10 mm rotational displacement would cause submaximal loading that would not disrupt the soft tissues within the complex. Anterior displacement of more than 3 mm is associated with ligament injury [9]. The normal range of axial rotation of the atlas on the axis is from 43 to 56° [10]. Based on an average atlas transverse diameter of 79.6 mm [11], lateral rotation to a displacement of 10 mm, corresponding to 15° of rotation, is well within the normal limits of rotation. This was deemed important as the purpose of this study was to assess the relative contributions of the bony and soft tissue structures within the C1-C2 complex and thus we wanted the mechanical analysis to evaluate the effect of our dissections, rather than disrupt the complex in any way. We wanted to test the stiffness of the C1-C2 complex before the point of failure and damage to the soft tissue structures. Load was applied such that the resultant displacement rate was 1 mm per second (1 mm/s). Mass applied (in grams) against displacement (in millimetres) was recorded. The displacement and force were measured by the testing rig itself (MACH-1). Therefore, no other device was required to measure the displacement or force.

Mechanical testing involved the following stages (AP and rotational testing occurred at each stage):

1. undissected specimen,
2. odontoid fracture: a 5 mm vertical incision was made anteriorly on either side of the anterior longitudinal ligament at the level of the base of the odontoid process to enable access to perform an osteotomy to simulate an Anderson and D’Alonzo type II fracture of the odontoid process. The osteotomy was performed using an osteotome and was carried out by the one of the authors (a clinician) in all cases,
3. facet joint capsules dissection,
4. ligamentum flavum dissection,
5. anterior longitudinal ligament dissection.

**Figure 4.**
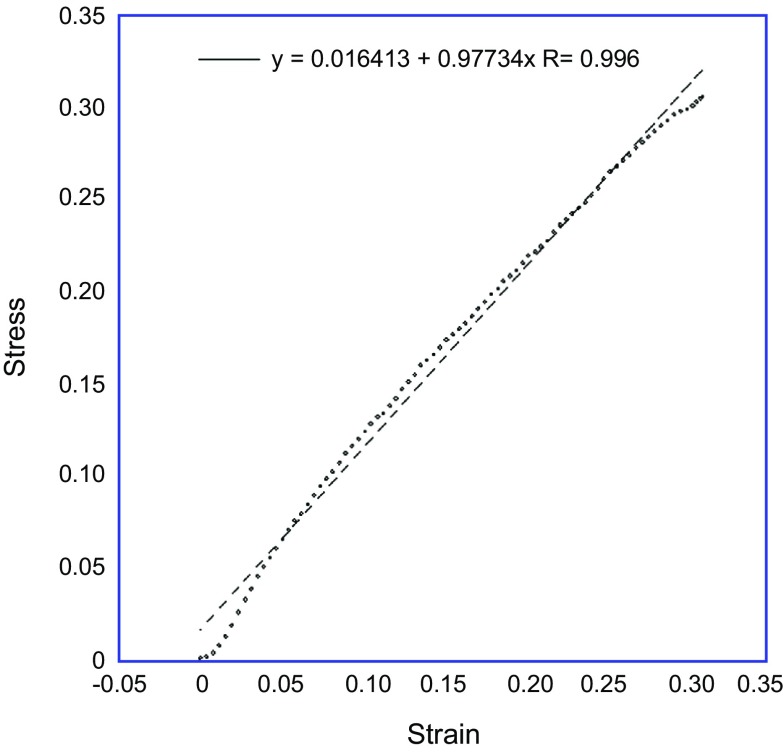
Graph showing Stress versus Strain in the undissected specimen in antero-posterior displacement biomechanical testing. Plotted line represents Young’s modulus, which is 0.977 MPa in this undissected specimen.

In a test specimen we found that the C1-C2 complex became very unstable after division of the anterior longitudinal ligament so we were unable to determine the contribution of the posterior longitudinal ligament in this study. We recorded load (in Newtons (N)) against displacement (mm). From the load and dimensions of the specimens we calculated stress (in Megapascals (MPa)). From the displacement and dimensions of the specimens we calculated strain. The calculation of stress and strain allowed the identification of Young’s Elastic Modulus through regression analysis (KaleidaGraph version 4.1, USA) for each stage of C1-C2 complex disruption. Figure 4 shows an example graph of stress plotted against strain, allowing the calculation of the Young’s Modulus using regression analysis.

Statistical analysis was performed using IBM SPSS Statistics Version 22 (IBM, New York, USA). Paired *T*-tests were performed to assess for statistically significant differences in Young’s Moduli between dissections.

## Results

**Figure 5.**
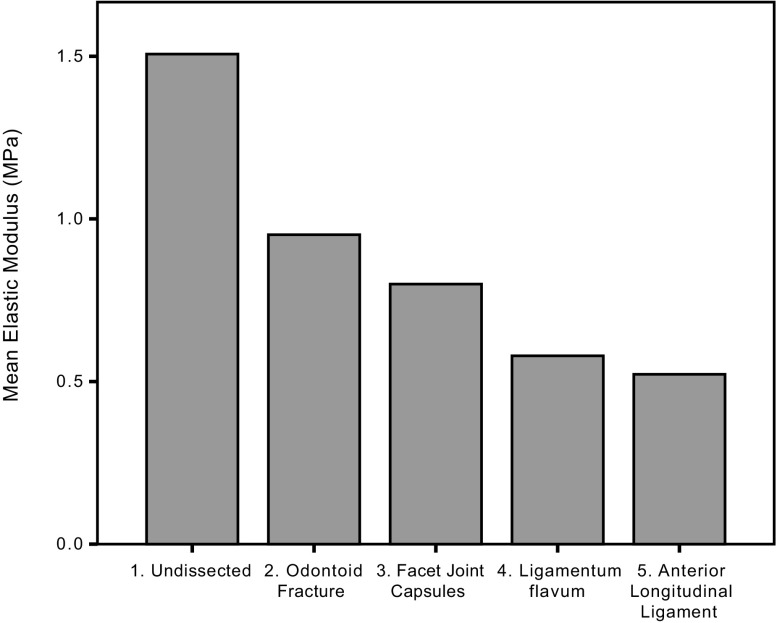
Graph showing successive reduction in the stiffness of the C1-C2 complex after successive division of the structures. Mean elastic modulus (MPa) displayed on *y*-axis. On *x*-axis: 1. Undissected specimen; 2. Specimen after fracture of the odontoid process; 3. Division of facet joint capsules; 4. Division of ligamentum flavum; 5. Division of Anterior Longitudinal Ligament.

In the undissected specimens the mean Young’s modulus was 1.51 MPa in anterior-posterior (AP) displacement. The mean Young’s modulus in AP displacement decreased by 37% when the odontoid process was fractured (*p* = 0.038, 95% confidence interval 0.04–1.07). Table 1 displays the resulting changes in stiffness of the C1-C2 complex as the odontoid process and soft tissue structures were divided. The table displays the changes in Young’s modulus when the C1-C2-complex was tested individually in AP displacement, left and right rotation. In the eight specimens the mean Young’s modulus in anterior-posterior displacement decreased proportionally (compared to the previous dissection) by the following percentages when the structures were divided: facet joint capsules (bilateral) 16%, ligamentum flavum 27%, anterior longitudinal ligament 10%. These differences were not statistically significant (*p* > 0.05). The differences in the elastic moduli in lateral rotation between the different dissections did not reach statistical significance (*p* > 0.05). Figure 5 displays the changes in stiffness in anterior-posterior displacement in graphical form. Table 2 displays an example of the forces (in Newtons) required to cause 3 mm of anterior-posterior displacement and 10 mm of lateral rotation from one of the eight specimens successively dissected and tested.

**Table 1.** Young’s Moduli (MPa) calculated from mechanical testing of the eight C1-C2 complexes at each successive dissection (mean and standard deviation).

	AP translation	Left rotation	Right rotation
Undissected	1.51 (±0.73)	0.43 (±0.46)	0.37 (±0.32)
Odontoid fracture	0.95 (±0.37)	0.12 (±0.13)	0.18 (±0.13)
Facet joint capsules	0.80 (±0.40)	0.10 (±0.14)	0.17 (±0.17)
Ligamentum Flavum	0.58 (±0.37)	0.06 (±0.07)	0.15 (±0.14)
Anterior longitudinal ligament	0.52 (±0.23)	0.04 (±0.02)	0.10 (±0.16)

**Table 2:** Force in Newtons (N) required to cause 3 mm of anterior-posterior (AP) displacement and 10 mm of lateral rotation in one of the eight specimens.

Dissection number	AP displacement force (N)	Left rotation force (N)	Right rotation force (N)
1	6.73	1.61	4.52
2	2.92	1.78	1.09
3	2.47	1.37	1.76
4	2.46	0.67	1.25
5	1.80	0.57	0.40

## Discussion

The odontoid process itself contributed to 37% of the overall stability of the C1-C2 complex in our study. Following an Anderson and D’Alonzo type II fracture of the odontoid process the ligaments and soft tissues accounted for the remaining stability. We have shown that the stability of the C1-C2 complex decreased after fracture of the odontoid process and then the stiffness successively decreased when the individual ligaments were divided. The C1-C2 complex in each specimen became very unstable after division of the anterior longitudinal ligament so we were unable to determine the contribution of the posterior longitudinal ligament from this study.

A limitation of our study was the number of specimens (*n* = 8). With a larger number of specimens we would have been able to determine the contribution of each ligament to the C1-C2 complex stiffness more accurately. We were also unable to determine the role of the cruciform ligament in stability of the C1-C2 complex as our cadaveric specimens did not always include the occiput. In addition, with more specimens we would have been able to perform posterior to anterior dissections to determine the contribution of the posterior longitudinal ligament (tectorial membrane) as, in this study, the C1-C2 complex was too unstable after dissection of the other structures to determine its stiffness. In our specimens once all the ligamentous structures except the posterior longitudinal ligament (tectorial membrane) were cut the C1-C2 complex was so unstable that we could not set up the rig to test the stiffness. When C2 was clamped in the testing rig and we positioned the MACH-1 testing device to test AP stability, C1 would fall away from C2 making it not possible to perform the mechanical testing without further supporting the construct. Further supporting the construct would make accurate measurements of the stiffness impossible, as they would have to take into account whatever was being used to stabilise the construct. In testing therefore there was an all or none effect as we were no longer able to test after sectioning the anterior longitudinal ligament. We are unable to infer from this whether this is the case in vivo, though, as in vivo the surrounding muscles will support the cervical spine to some extent also.

Currently, the management of Anderson and D’Alonzo type II fractures of the odontoid process remains controversial. Operative treatment is generally recommended for older patients, in fractures with posterior displacement and when the displacement of the fracture is greater than 4–6 mm [6]. Biomechanical studies, until recently, had not looked specifically at the role of the atlantoaxial ligaments after odontoid process fractures.

Crawford et al. [12] performed an interesting biomechanical study on C2 fractures. They used fresh-frozen cadaveric cervical specimens and assessed the biomechanical stability of the spine after simulating transverse-apical-alar ligament disruptions, type II odontoid process fractures and odontoidectomies. Although in their study they simulated type II odontoid process fractures and assessed their biomechanical stability they did not look at the stability after C2 fractures when different ligaments were disrupted in turn [12].

McCabe et al. [13] recently published a biomechanical cadaveric study assessing the role of the soft tissues in stabilising the spine after type II odontoid process fractures. They dissected 10 fresh frozen cadaveric spines and assessed the biomechanics of the spine after performing an odontoid process osteotomy from a posterior incision, which involved dissecting the tectorial membrane and vertical element of the cruciate ligament to gain access to perform the osteotomy. This was followed by sequential sectioning of the soft tissue restraints in two groups. Sectioning protocol 1 had unilateral then bilateral sectioning of the facet joint capsules. Sectioning protocol 2 had sectioning of the anterior longitudinal ligament (ALL) then the right facet joint capsule. Both groups were then tested in “the complete injury state” of having both facet joint capsules and the ALL sectioned. They found a stepwise increase in axial rotation with every soft tissue structure divided. Interestingly though they only found an increase in anterior-posterior translation in “the complete injury state”. Their study differed from ours in its methodology. They tested the amount of displacement that occurred after a fixed 10 N force was applied [13]. Our study assessed the force required to cause 3 mm of anterior displacement and 10 mm of rotational displacement. We chose this method of testing because it would cause submaximal loading that would not disrupt the soft tissues within the complex. Our study supports their findings that the C1-C2 complex after odontoid fracture becomes increasingly more unstable with ligament damage. Their study was able to find significant differences in stiffness in axial rotation between dissections whereas ours was not. We believe this is due to our methodology of only testing 10 mm rotational displacement. This may not sufficiently stress the C1-C2 complex enough to determine differences between the dissections in axial rotation. Their study and ours highlight the fact that surgeons should consider the role of the ligaments in maintaining stability after odontoid fractures and magnetic resonance imaging (MRI) may have a role in detecting ligament damage.

Non-operative management is currently preferred in patients with type II odontoid process fractures, which are less than 5 mm displaced on CT imaging. Non-operative management may consist of bracing or halo-ring external fixation. Halo devices carry a risk of respiratory problems and, therefore, non-operative management may not always be a benign method of treatment [2, 6].

A cadaveric biomechanical study found that MRI could detect anterior and posterior longitudinal ligament injuries reliably. MRI was less reliable at detecting capsular or ligamentum flavum injuries in this study [14]. We would like to propose that patients with type II odontoid process fractures that are displaced less than 5 mm and are being considered for non-operative management should undergo magnetic resonance imaging (MRI) of their cervical spine to assess the status of the ligaments and soft tissues, if MRI facilities are readily available. If the MRI determines that the ligaments are intact and the fracture is minimally displaced, non-operative management may be continued. If the MRI detects that there is associated anterior or posterior longitudinal ligament disruption the fracture is likely to be unstable and we would suggest operative management.

In conclusion, we have found that the odontoid process itself may account for 37% of the stiffness of the C1-C2 complex and that soft tissue structures account for further resistance to movement. We suggest magnetic resonance imaging of the soft tissues in the acute setting of a minimally displaced odontoid process fracture to plan management of the injury. If the MRI determines that there is ligament injury in addition to the odontoid fracture it is likely that the fracture is unstable and we would suggest operative management.

## Conflicts of interest

ORB, JB and MS declare no conflicts of interest.
